# Distribution of *EGFR* and *KRAS* Mutations in Patients with Surgically Resected Non-Small Cell Lung Cancer from Southern Italy: Real-Life Data from a Single Institution and Literature Review

**DOI:** 10.3390/cancers17050730

**Published:** 2025-02-21

**Authors:** Michele Piazzolla, Paola Parente, Flavia Centra, Federico Pio Fabrizio, Marco Donatello Delcuratolo, Antonella Centonza, Concetta Martina Di Micco, Mario Mastroianno, Francesco Delli Muti, Fabiola Fiordelisi, Gianmaria Ferretti, Paolo Graziano, Lucia Anna Muscarella

**Affiliations:** 1 Unit of Thoracic Surgery, IRCCS Casa Sollievo della Sofferenza, 71013 San Giovanni Rotondo, Italy; m.piazzolla@operapadrepio.it (M.P.); gianmaria.ferretti@asl.taranto.it (G.F.); 2 Unit of Pathology, IRCCS Casa Sollievo della Sofferenza, 71013 San Giovanni Rotondo, Italy; paolaparente77@gmail.com (P.P.); fabiolafiordelisi@gmail.com (F.F.); paolo.graziano@uniroma1.it (P.G.); 3 Laboratory of Oncology, IRCCS Casa Sollievo della Sofferenza, 71013 San Giovanni Rotondo, Italyfedericopio.fabrizio@unikore.it (F.P.F.); f.dellimuti@operapadrepio.it (F.D.M.); 4 Unit of Oncology, IRCCS Casa Sollievo della Sofferenza, 71013 San Giovanni Rotondo, Italy; donatello.m.delcuratolo@gmail.com (M.D.D.); doctor.dimicco@gmail.com (C.M.D.M.); 5 Scientific Direction, IRCCS Casa Sollievo della Sofferenza, 71013 San Giovanni Rotondo, Italy; m.mastroianno@operapadrepio.it

**Keywords:** NSCLC, surgical resection, early stages, molecular markers, *EGFR*, *KRAS*

## Abstract

Background/Objectives: The identification of driver mutations in NSCLC such as those in the *EGFR* and *KRAS* genes has revolutionized the understanding and management of many lung cancer patients and has opened up a new scenario in the early disease stages in terms of therapeutic options (EGFR) and prognosis (KRAS). Data on prevalence rates and disease stage distributions of *EGFR* and *KRAS* mutations in surgically resected NSCLC are growing, but in Southern Italy, estimation is limited, since upfront *EGFR* testing in early-stage adenocarcinoma has been only recently introduced according to the current guidelines in clinical practice, whereas *KRAS* screening is usually uninvestigated in resected NSCLC. In this real-life study of a single institution in the Apulia Region, we provide an overview of the epidemiological distribution of *EGFR* and *KRAS* mutations in patients in Southern Italy with resected NSCLC, highlighting their prevalence, clinical significance, and correlation with demographic and pathological factors. A literature review was also performed to compare our findings with the most recent available data from the screening of Italian cohorts of advanced and surgically resected NSCLC patients. Methods: Data from 149 patients coming from Southern Italy with surgically resected NSCLC were retrospectively collected over a period of 16 years. *EGFR* and *KRAS* mutation screenings were performed and correlated with clinical and pathological data. Results: In total, 24 out of 149 NSCLC (16%) patients harbored an *EGFR* mutation. Exon 19 deletions and missense p.L858R mutations of the *EGFR* gene have quite similar frequencies (46%) and were more observed in never smokers (*p* < 0.001) and female (*p* < 0.001) patients with the adenocarcinoma histotype. *KRAS* gene mutations were detected in 31.5% of cases, with missense p.G12C (32%), p.G12V (28%), and p.G12D (17%) mutations as the most frequent ones. Neither *EGFR* nor *KRAS* mutational status were found to impact overall survival (OS) in our study cohort. Conclusions: Our findings improve the understanding of lung cancer genetics in a small and homogeneous area of Southern Italy and guide future research. The *EGFR* and *KRAS* mutations in NSCLC resected patients from Southern Italy showed a global similar incidence compared to other recently described Italian cohorts of advanced and early-stage NSCLC, with a higher frequency of exon19 *EGFR* deletions. No prognostic impact was observed for both *EGFR* and *KRAS* status, but additional investigations on a larger cohort are needed.

## Simple Summary

Lung cancer is a leading cause of cancer-related deaths, with non-small cell lung cancer (NSCLC) being the most common type. Specific molecular signatures, such as *EGFR* and *KRAS* mutations, are taking on a key role in understanding and treating this disease, also in the early stages. However, little is known about the prevalence of these mutations in resected NSCLC patients from Southern Italy, as routine molecular testing of *EGFR* has only recently been adopted in the early-stage non-squamous histotype. This study aims to describe the epidemiology of *EGFR* and *KRAS* mutations in resected NSCLC cases from a single institution in Southern Italy, their links to patient characteristics, and their prognostic impact. These results may improve our understanding of the lung cancer molecular profile/signature in this specific geographical area and guide future research.

## 1. Introduction

Lung cancer represents the leading cause of cancer death in the world [1] and is a highly heterogeneous pathology in which various histological subgroups have been identified, each with specific cytoarchitectural and molecular characteristics [2]. Among these, non-small cell lung cancers (NSCLCs) are the most representative group (approximately 85% of all lung tumors) and adenocarcinoma (ADC) is the most frequent histotype (more than 50% of all NSCLCs) [1]. Approximately 30% of patients with NSCLC are candidates for radical surgery (stages I–IIIA) and show a generally better prognosis than the advanced ones [3]. However, even in early-stage patients, disease-free survival (DFS) does not exceed 5 years due to the extremely aggressive nature of the disease [3].

For many years, in the completely resected patients with II–IIIA disease stages, the adjuvant treatment of choice was represented by platinum-based chemotherapy, which, however, offers limited benefit [4]. More recently, the effectiveness in the adjuvant setting of biological therapies already used for years in the treatment of advanced stage NSCLC patients has been proven and epidermal growth factor receptor (*EGFR*) molecular screening has been introduced in the adjuvant setting [5]. In particular, the ADAURA study demonstrated a significant benefit in terms of disease free-survival (DFS) and overall survival (OS) in the use of the EGFR receptor tyrosine kinase inhibitor osimertinib in the adjuvant setting, in resected lung adenocarcinoma patients with activating mutations in exons 19 and 21 of the *EGFR* gene [6]. By consequence, the AIOM (Italian Association of Medical Oncology) guidelines recently recommended the screening of *EGFR* in early resected IB–IIIA patients to select candidates for osimertinib treatment in the adjuvant setting (https://www.aiom.it (accessed on 10 February 2025)).

Activating *EGFR* mutations represent the most common druggable target in metastatic NSCLC with non-squamous histology and show a well-documented incidence, varying between 10% in the European population and more than 64% in the Asian population [2,7,8,9,10]. By contrast, given the recent introduction of *EGFR* mutation screening in the clinical practice for early-stage resected NSCLCs, data on the prevalence and distribution of *EGFR* mutations in these patients are only recently being collected, with very limited world-wide studies [11,12]. Furthermore, many published studies have been conducted on pre-selected cohorts of NSCLC patients, suffering from consequent bias in terms of the frequency of *EGFR* mutations or in other driver oncogenes considered as potential prognostic factors in early-stage NSCLC such as the Kirsten rat sarcoma viral oncogene homolog (*KRAS*) gene [13,14].

*KRAS* mutations are present in approximately 20–30% of lung adenocarcinomas, are more prevalent in Western populations, and are strongly linked to smoking history [2,15]. Despite its initial challenges of being “undruggable”, several inhibitors that specifically bind KRAS-G12C have been developed and investigated in clinical trials, with sotorasib becoming the first treatment to gain approval for adults with stage IV NSCLC harboring a KRAS-G12C mutation as a second-line therapy [15,16,17,18,19]. Therefore, there is still disagreement regarding the predictive significance of the *KRAS* mutational status in early-stage NSCLC [20]. Indeed, current clinical guidelines do not recommend testing for *KRAS* mutations in the resectable stages of NSCLC due to the lack of agreement regarding its implications on prognosis (https://www.iaslc.org; https://www.aiom.it (accessed on 10 February)) [21].

Long-term data on the course of the disease in resected early-stage NSCLC patients with *EGFR* and *KRAS* mutations are also limited, although these represent extremely important data in the context of the recently approved adjuvant anti-EGFR therapy in stage IB–IIIA NSCLC after radical resection cited above [6]. Moreover, even if the prevalence of *EGFR* and *KRAS* mutations in the Italian population has been reported [22], very few studies were assessed in the early stages and they were restricted to certain geographical areas, and data about Apulia (Southern Italy) is missing.

By performing molecular profiling at the Casa Sollievo della Sofferenza Hospital (San Giovanni Rotondo, Foggia, Apulia), our retrospective cohort study provides a unique real-world dataset to assess the frequency and distribution of mutations in lung cancer driver genes, such as *EGFR* and *KRAS*, in early-stage (stage I–IIIA) NSCLC patients from Southern Italy undergoing radical surgical resection.

## 2. Materials and Methods

### 2.1. Study Population and Clinical Parameters

This study involves a total of 149 retrospectively enrolled patients diagnosed with Stage I–IIIA NSCLC, who underwent curative radical surgery during the period between January 2007 and December 2023 at the Thoracic and Pulmonary Surgery Department of the Casa Sollievo della Sofferenza Hospital in San Giovanni Rotondo (FG), in Southern Italy, Apulia Region. All patients included in this study underwent anatomical resection of the lung parenchyma with an open or minimally invasive approach (VATS or robotic) or an atypical resection. For all patients, clinical and pathological information that included the Tumor–Node–Metastasis (TNM) staging system, age at diagnosis, type of surgery, histology, and smoking history were collected in collaboration with the Units of Pathology, Oncology, and Thoracic Surgery of the hospital. Patients were classified as “current smokers” if they smoked up to one month before diagnosis, “former smokers” if they quit smoking at least one month before diagnosis, and “never smokers” if they smoked less than one pack/year during their lifetime. Exclusion criteria were the presence of multiple primitive tumors and non-NSCLC histology.

Patients enrolled in this study were treated according to standard clinical practice and the guidelines by the Italian Society of Medical Oncology (AIOM, www.aiom.it). The clinical–pathological findings and the biological samples used in this study are compliant with the Declaration of Helsinki, after Local Ethics Committee Approval and with the informed consent of each patient for molecular screening (nos. 89/CE and 171/CE).

### 2.2. Biological Sample Collection

NSCLC tissues from primary tumors (surgically resected, with >50% of tumor cells) from each patient were collected as sections (10 μm thick) from Formalin-Fixed Paraffin-Embedded (FFPE) blocks. All cases were revised by two expert pathologists (P.P. and P.G.) according to the current World Health Organization (WHO) classification of tumors by IARC (https://publications.iarc.fr (accessed on 10 January 2010)), and one FFPE block was selected for each patient to extract DNA.

### 2.3. Molecular Analysis

Four-micrometer-thick tissue sections were cut and stained with hematoxylin and eosin (H&E), after their fixation in 10% buffered formalin for 24–48 h. DNA was extracted after slide microdissection using the GeneRead FFPE kit (Qiagen, Hilden, Germany) and quantified with the Qubit dsDNA BR assay kit (Life Technologies, Carlsbad, CA, USA) on a Qubit fluorimeter (Thermo Fisher Scientific, Waltham, MA, USA). *EGFR* and *KRAS* mutations were searched for in each biological sample using both sequencing techniques (pyrosequencing or DNA-based next generation sequencing, NGS, on the Ion Torrent platform (Thermo Fisher)) or hot spot techniques (ddPCR), depending on the quality of DNA.

For the NGS library preparation and sequencing, a minimum of 30 ng of DNA was subjected to amplification using the Colon and Lung Cancer Research Panel v2 (Ampliseq™, Life Technologies) and the Ion Ampliseq™ Library kit 2.0. For each quantified library 6–8 pM was pooled and amplified by emulsion PCR and the obtained templates were enriched and successively loaded on an Ion 520™ chip and sequenced on Ion Gene Studio S5 (Thermo Fisher Sc.). Sequencing data were processed using the standard Ion Torrent Suite Software (v 5.16) running on the Torrent Server. Raw signal data were analyzed using Torrent Suite using an analysis pipeline which includes read alignment to the human genome 19 reference (hg19), quality control of mapping, and coverage analysis. Following data analysis, the annotation of single nucleotide variants, insertions, and deletions was performed by the Ion Reporter Server System (Thermo Fisher Sc.). For *EGFR* and *KRAS* mutations, only pathogenic mutations, assessed by reference databases, were included.

The resulting DNA samples which were inadequate for NGS analysis were analyzed by droplet digital PCR on a Droplet Digital™ PCR System (Bio-Rad, Hercules, CA, USA) with appropriate commercial primers and probes (https://www.bio-rad.com) to identify mutations on exon 2 of the *KRAS* gene (codons 12 and 13) and *EGFR* gene (exon 19 deletions and p.L858R). Droplets were generated using a Droplet Generator and then amplified in a C1000 Touch™ deep well Thermal Cycler (Bio-Rad). After cycling, the 96-well plate was placed into the QX200 Droplet Reader (Bio-Rad) where the droplets of each sample were analyzed sequentially, and the fluorescent signals of each droplet were measured individually by a detector. Data were analyzed with the Quanta Soft analysis software, version 1.7.4 (Bio-Rad). In negative samples, the presence of an *EGFR* mutation was assessed using the Therascreen EGFR Pyrosequencing Kit on Pyromark Q24 (Qiagen).

### 2.4. Statistical Analysis

The clinicopathological characteristics of patients will be reported as medians or in frequencies and percentages for continuous and categorical variables, respectively. The overall survival time (OS) will be defined as the time between surgery and death.

All statistical analyses were performed using the R software version 4.3.0. Survival curves were estimated using the Kaplan–Meier method with the survfit() function from the survival package (version 3.5). The *p*-value for group comparisons in the survival curves was calculated using the log-rank test, automatically performed through the ggsurvplot() function from the survminer package (version 0.4.9). Furthermore, as always for the study of survival, a univariate Cox regression analysis was also performed, from which the hazard ratio, 95% confidence intervals, and *p*-values (calculated via Cox’s test) were derived; these calculations were carried out using the coxph() function from the survminer package.

Group comparisons for *EGFR* and *KRAS* were conducted using the gtsummary package (version 1.7.2) with the tbl_summary() function, applying either a t-test or chi-squared test for intergroup comparisons.

## 3. Results

### 3.1. Patient Cohort

A total of 114 (77%) of the 149 patients enrolled were males, and the mean age was 67 (range 40–85). Only 25% (37 cases) were never smokers, 37% (55 cases) were former smokers, and 38% (57 cases) were current smokers. A total of 83 out of 149 NSCLCs (83%) were primary adenocarcinomas (ADCs), 13% (19/149) were squamous cell carcinomas (SqCCs), (19/149), and the remaining 4.7% (7/149) consisted of adenosquamous (ASC) or less frequent histotypes. All patients enrolled in this study come from Southern Italy (Apulia Region). Both *EGFR* and *KRAS* mutations were determined in all samples; the distribution of patients was uneven across the TNM stages of the 8th edition, with fewer patients in stage IIA (4.7%) and more patients in stage IA and IB (28.9% and 26.2%), IIB (21.5%), and IIIA (18.8%), (Table S1).

### 3.2. Distribution of EGFR Mutations in Early-Stage NSCLC

**Figure 1:**
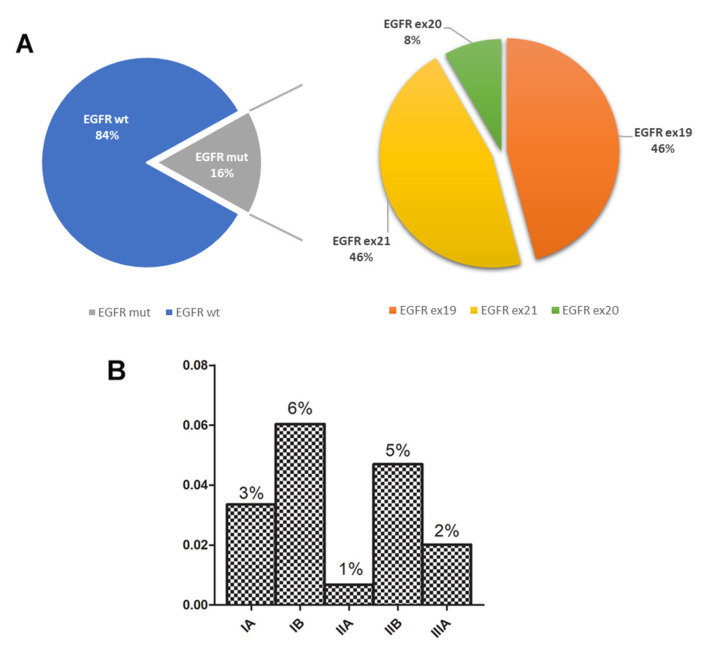
*EGFR* mutation prevalence and subtypes across NSCLC disease stages. (**A**) Prevalence of *EGFR*-mutated cases across the TNM stages (TNM 8th edition). (**B**) Distribution of different *EGFR* mutations identified in all surgical specimens.

A total of 24/149 cases (16%) harbored an *EGFR* mutation, with a decreasing prevalence in IB (38%), IA (21%), and IIB (29%) patient stages (Table 1 and Figure 1B).

The *EGFR*-mutated patients (16%) harbored a similar frequency of exon 21 (46%) and ex19 mutations (46%), whereas few patients showed mutations in exon 20 (8%) (Figure 1A). The characteristics of the *EGFR*-mutated cohort are outlined in Supplementary Table S1. The clinical, molecular, and histological parameters among the patient cohort were also compared (Table 2). *EGFR* mutations were more frequent in females than in males (*p* < 0.001) and in never smoker patients (*p* < 0.001), but were randomly distributed among the disease stages. A total of 83% (20/24) of mutations were identified by sequencing methodologies, allowing a more precise characterization of the mutation type at a single nucleotide level.

### 3.3. Distribution of KRAS Mutations in Early-Stage NSCLC

**Figure 2:**
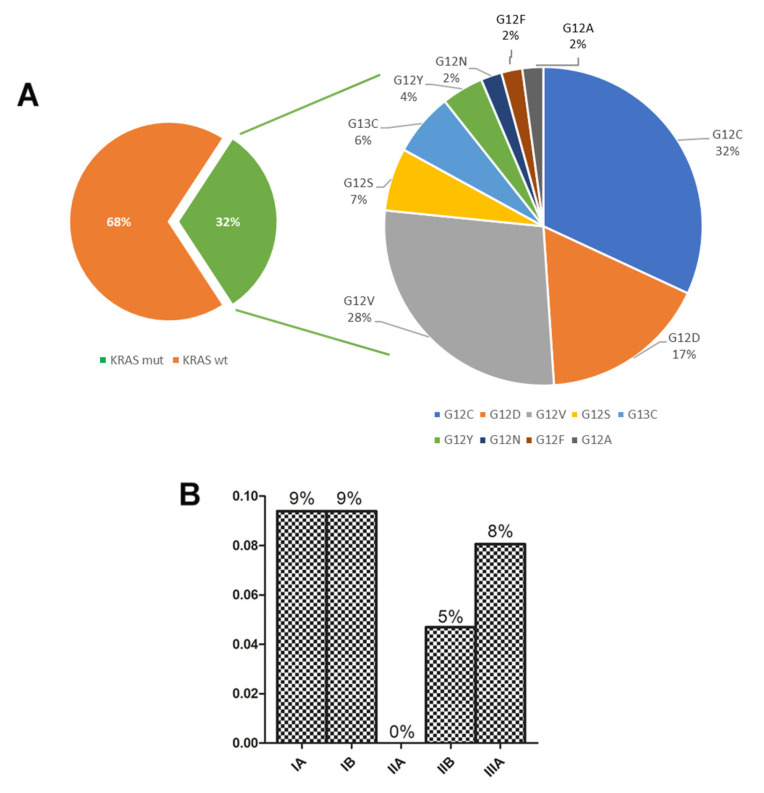
*KRAS* mutation prevalence and subtypes across NSCLC disease stages. (**A**) Prevalence of *KRAS* mutated cases across the TNM stages (TNM 8th edition). (**B**) Distribution of different *KRAS* mutations identified in all surgical specimens.

A total of 47/149 (31.5%) of patients harbored a *KRAS* mutation, with the stages IA (30%) and IB (30%) as the most prevalent ones (Table 1 and Figure 2B). The characteristics of the *KRAS*-mutated cohort are outlined in Supplementary Table S2. *KRAS* mutations were randomly distributed among disease stages but are more frequent in SqCC than in ADC histology (*p* < 0.03). The sequencing methodology approach allowed us to identify more *KRAS* mutations than the hot spot approach (Table 3). The most common subtype was p.G12C (32%), followed by p.G12V (28%) and p.G12D (17%), (Figure 2A).

### 3.4. Prognostic Impact of EGFR and KRAS Mutation Status

**Figure 3:**
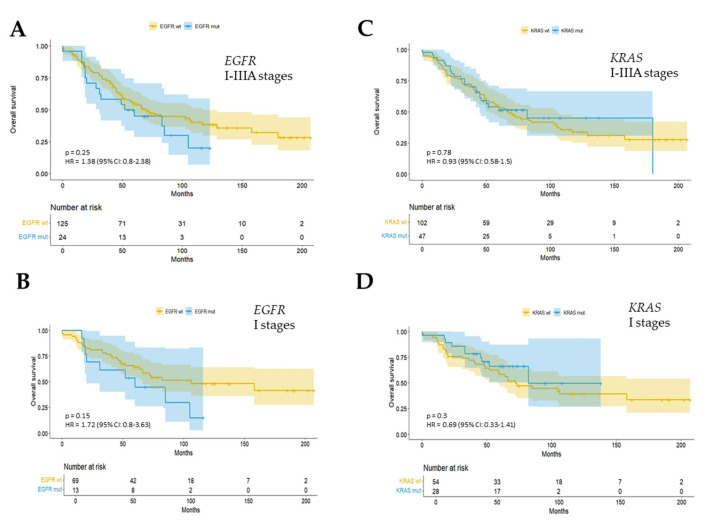
Overall survival in all patients (I–IIIA stages) according to *EGFR* (**A**) and *KRAS* (**C**) mutation status. Overall survival in stage IA–B patients according to *EGFR* (**B**) and *KRAS* (**D**) mutation status. OS, overall survival; *EGFR*, epidermal growth factor receptor; wt, wild type; mut, mutated.

To evaluate the prognostic impact of the *EGFR* and *KRAS* mutation status, the overall survival (OS) in the 149 patients, with a median follow-up of 8 ± 4.2 years, was analyzed. A total of 82/149 patients were in stage IA-B, with a median follow-up of 8.5 ± 4.2 years. No association of *EGFR* or *KRAS* mutation and OS was observed either in the entire cohort (stages I–IIIA patients) or in patients with I disease stages (IA–IB) (Figure 3).

## 4. Epidemiology of *KRAS* and *EGFR* Mutations in Italy and Southern Italy

Heterogeneous frequencies and different distributions of *EGFR* and *KRAS* alterations have been reported in many study populations, due to discrepancies in terms of sensibility and specificity among different generations of sequencing technologies which are gaining ground [23].

*EGFR* mutations were identified in 17% of ADCs from surgically resected patients recruited who underwent surgical resection at the Division of Thoracic Surgery of the University of Modena and Reggio Emilia, whereas *KRAS* mutations were found in 42% of patients (mainly p.G12C, p.G12V, and p.G12D). *EGFR* mutations were also strongly related to the never smoker status (*p* = 0.002) and the female gender (*p* = 0.05) [24]. No significant correlation among *KRAS* mutation, gender, or smoking habit was found. A high ratio of *EGFR* exon 21/exon 19 mutations was reported in this study, in disagreement with that commonly reported in similar and larger NSCLC studies [25].

The epidemiology of *EGFR* and *KRAS* mutations was also investigated by Normanno and Colleagues in order to clarify their prognostic impact. The study was conducted to establish the *EGFR* mutation profile of 3567 advanced NSCLC patients from a Southern Italy cohort. *EGFR* mutations were identified in 14.6% of cases and were mainly associated with the ADC histology (15.7%), female gender (25.2%), and non-smoking habit (32%). The identified *EGFR* activating mutations were mainly located at exon 19 (9.4%), whereas 4.6% of mutations clustered at exon 21 [26].

A similar epidemiologic analysis was performed by Rachiglio *and Colleagues* on a cohort of 133 advanced and metastatic NSCLC patients recruited from seven Southern and Central Italy hospitals and treated with first-line therapy EGFR tyrosine kinase inhibitors (TKIs). All patients were profiled for *EGFR* and *KRAS* mutations by the NGS approach using targeted panels. The distribution of somatic mutations was roughly consistent with those already reported. *EGFR* exon 19 deletion was identified in 83/133 patients (62.4%), whereas 39/133 (29.3%) had an *EGFR* p.L858R point mutation and 11/133 (8.3%) harbored different *EGFR* mutations, which are mostly present in women (92/133; 69.2%) and never smokers (81/133; 61.4%). Moreover, *KRAS* mutations were detected in 14/133 cases, and this suggests its predominance as a consistent part of the total number of mutations detected, different to *EGFR* [27].

Results from a prospective study on *EGFR* and *KRAS* mutations were reported by Colombino M et al. in order to screen more than 1000 surgically resected lung ADC cases from January 2011 to July 2016 in Sardinia. The investigation of the recurrent *EGFR* alterations led to a similar incidence in other Caucasian countries (13%), including exon 19 deletions and p.(L858R) missense aa changes (38% and 29% of all those observed, respectively) which were found in females and non-smokers [28]. Nevertheless, *KRAS* mutations were identified in males and in smokers at 22% and are a hallmark of being mutually exclusive to *EGFR*. Co-occurring mutations in key driver genes were identified, only in very few cases, as a possible mechanism of primary drug resistance [28]. Likewise, Passiglia F and Colleagues collected molecular and prognostic data about *KRAS* mutations from 50 medical oncology/pathology divisions across several Italian regions. In terms of results, *KRAS* mutations did not show any significant discrepancies between geographical areas (69% north versus 63% south of Italy) in a large group of metastatic, non-squamous NSCLCs across all disease stages [29].

A more recent audit performed on 1100 advanced NSCLC patients from the ATLAS knowledge-based database (including Southern and Northern Italy Centers) was proposed by Malapelle U and Collaborators. The study aimed of clarify the epidemiological landscape of genetic alterations and revise the diagnostic tool for the management of patients with NSCLC. In about 50% of NSCLC patients subjected to molecular screening, targetable drivers were found in *EGFR* (13.8%) and *KRAS* (26.5%). *EGFR*-mutated patients were more distributed, ranging from ages 60 to 80 years (66.2%) with a major preference for female cases (64.5%), ADC histology (91.6%), and non-smokers (52.1%). Secondly, the detection of *KRAS* mutations followed a heterogenous distribution among gender (female 30.3% versus male 69.7%) and tobacco habits (former > 10 p/y in 52.9%), with an opposite trend compared to *EGFR* status, although the elderly population and ADC remain in common with this latter one [22].

A more recent Italian multicenter experience was conducted by Pepe et al. to provide an epidemiological and molecular landscape about predictive biomarker selection and validation in NSCLC Italian patients [30]. The analytical performances of molecular tests in more than 20 Italian research/diagnostic centers (differently distributed as follows: 58.4% in Northern, 20.8% in Central, and 20.8% in Southern Italy) were tested. Hotspot mutations at exon 2 (p.G12C) of *KRAS* and deletions at exon19 (p.E746_A750del) of *EGFR* for all participating Institutions by next generation sequencing (NGS), RT-PCR (Real-Time Polymerase Chain Reaction), NGS plus RT-PCR, and pyrosequencing systems were identified at the similar and just reported incidence. In this context, the researchers suggested that the implementation of engineered controls (e.g., artificial cell lines harboring genomic alterations) in harmonized procedures could represent a valuable solution to improve the technical sensitivity and efficiency of diagnostic workflows in preclinical and clinical practice, regardless of different geographical distributions of molecular and genomic diagnostic laboratories [30].

## 5. Discussion

The field of NSCLC treatment has undergone significant transformation as a result of molecular profiling in patients. In our study, about 48% of the 149 patients had a genetic change in one of the two *EGFR* and *KRAS* genes. This ratio is consistent with the previous findings on the epidemiology of NSCLC druggable mutations, where almost half of patients with lung adenocarcinoma had *EGFR* or *KRAS* mutations [22]. *EGFR* mutations in our cohort appear to be more frequent than those reported in the European population [24,26,27,31,32,33]. This might be related to the genetic traits of the group we are studying, as we are the first to report on the population of patients with surgically resected NSCLC with high genetic homogeneity restricted to the Apulia Region (Southern Italy). Few patients showed mutations in exon 20 (8%). The identified *EGFR* mutations in 16% of early-stage NSCLC cases were mainly associated with ADC histology (96%), the female gender (63%), and the non-smoking habit (63%), as was reported in large studies [22,34]. No significant association was found between *EGFR* mutation and OS of patients, even considering early stages I. These data were not in line with other previous published works, where a clear strong association of pathological stage I resected NSCLC patients with longer OS and progression-free survival (PFS) was reported [33]. Differences in OS between patients with *EGFR* exon 21 p.(L858R) mutations and those with exon 19 deletions were not investigated, due to the small cohort and low number of *EGFR* mutation events. Nevertheless, it must be considered that any prognostic data about the role of the *EGFR* status should be re-evaluated and integrated with predictive data considering the recent introduction of osimertinib treatment in the adjuvant setting in early-stage resected ADC with EGFR mutations [6].

By contrast, *KRAS* mutations tend to be associated with smoking habits, whereas never smokers showed a decreased risk of *KRAS* mutations, as observed in the past examined populations [35]. Additionally, in our series, the most common *KRAS* alterations were p.G12C (32%), p.G12V (28%), and p.G12D (17%), and other p.G12/G13 mutations (23%) were similar to that previously reported both in surgically resected early-stage NSCLC and the advanced ones [24,32].

*KRAS* p.G12C is a prevalent gene mutation found in NSCLC, which results in a substitution of cysteine (C) for glycine (G) at the 12th amino acid residue of the *KRAS* gene. This alteration activates the *RAS/MAPK* signaling pathway and sustains proliferation signaling, thereby facilitating the growth and spread of tumor cells [19]. The *KRAS* p.G12C mutations harbor more aggressive clinicopathologic and genomic features than other KRAS-mutant tumors and were associated with worse DFS after the complete resection of stage I–III lung adenocarcinoma, a correlation that was not investigated in our cohort [36]. Nine different *KRAS* mutations affecting codons 12 and 13 of exon 2 have been identified in NSCLC, as has been widely reported [http://cancer.sanger.ac.uk/cosmic (accessed on 20 January 2025)]. Some evidence seems to indicate that the different amino acid substitutions have distinct binding affinities for *KRAS* downstream effector molecules. In vitro analysis of cell lines carrying different *KRAS* codon subtypes showed that different amino acid substitutions in *KRAS* could activate different transcriptional programs affecting the prognosis or therapeutic approach [37]. In early-stage NSCLC, however, while several studies have shown that *KRAS* mutations negatively influence the prognosis [38,39], others have shown no significant effect [40], as confirmed in our cohort.

The small cohort investigated is the primary limitation of our study; this only depends on the availability and quality of sample tissue for testing and the gradual deployment of such analysis in clinical practice using different methodologies. Our work’s strengths, however, are the early-stage resected cases drawn from actual clinical practice and the genetic homogeneity of the population analyzed.

Going forward, much remains to be explored on the role of *KRAS* mutations in early NSCLC. In the age of precision medicine, more extensive studies will contribute toward the detailed level clinical data that are required for future pooled analyses of prognosis assessments that can guide clinical decisions.

## 6. Conclusions and Future Directions

Understanding the distribution and interaction of EGFR and KRAS mutations is essential for developing personalized treatment strategies and improving outcomes for patients with resected NSCLC. Current evidence suggests that targeted therapies might be effective in patients with resectable EGFR-mutant NSCLC. In any case, we must stress that screening for *EGFR* mutations in early-stage non-small cell lung cancer is merely the first step toward improving patient care. The relevance of *KRAS* mutations in early NSCLC is still largely unknown, which supports the usefulness of *KRAS* screening in this disease context to better explore the prognostic and predictive role of this marker in this disease setting. Many published data add to a growing body of evidence that suggests that lung cancer is composed of multiple genetic and clinical subgroups that are linked to distinct clinical disease behaviors. This emphasizes how crucial thorough tumor characterization is for clinical practice and future research at every stage of the disease. Since tumor cell architecture plays a crucial role in determining the tumor’s aggressiveness, it is best to link the detailed molecular profiling, if possible, with the tumor’s histopathologic features to prevent misinterpretation of the molecular findings. When assessing the potential utility and viability of anticipating tumor profiling in an early disease setting, several potential benefits must be considered, even though there is a lot of data only available in the metastatic setting. These include the increased likelihood of obtaining successful samples and the sensitivity in actionable target detection, which could improve clinical outcomes. Last but not least, the typically limited tissue available for the analysis, the short turnaround time for tumor profiling results, and the crucial coordination and alignment needed by the lung cancer multidisciplinary team may make the molecular profiling of lung cancer in the neoadjuvant setting particularly difficult. In any case, this information should be incorporated into the new artificial intelligence (AI) method that, when combined with liquid biopsy, will enable the acquisition of a dynamic picture of the progression of the disease in both advanced and early stages of lung disease. The goal is to maximize therapeutic efficacy while lowering treatment toxicities and expenses for patients with onco-gene-addicted NSCLC receiving systemic therapies. In this context, the epidemiological insight underscores the importance of tailoring therapeutic strategies to the genetic landscape of NSCLC, ensuring optimal care for different patient populations.

## Figures and Tables

**Table 1:** Distribution of *EGFR* and *KRAS* mutations across TNM lung disease stages.

Disease Stage	All Cases, n = 149	*EGFR*-Mutated, n = 24	*KRAS*-Mutated, n = 47
Stage IA	43 (29%)	5 (21%)	14 (30%)
Stage IB	39 (26%)	9 (38%)	14 (30%)
Stage IIA	7 (5%)	1 (4%)	0 (0%)
Stage IIB	32 (21%)	7 (29%)	7 (15%)
Stage IIIA	28 (19%)	3 (13)	12 (26%)

**Table 2:** Distribution of *EGFR* mutations according to patients’ features.

	Overall (n = 149)	*EGFR* wt ^1^ (n = 125)	*EGFR* mut ^2^ (n = 24)	*p*-Value
**Age at Diagnosis (mm ± SD)**	67.8 ± 8.8	67.7 ± 8.9	68.4 ± 7.9	0.73
**Sex**				**<0.001**
Female	35 (23%)	20 (16%)	15 (63%)	
Male	114 (77%)	105 (84%)	9 (38%)	
**Type of Surgery**				0.78
Anatomic resection *	109 (73%)	92 (74%)	17 (71%)	
Atypical resection	40 (27%)	33 (26%)	7 (29%)	
**R**				0.81
0	144 (97%)	121 (97%)	23 (96%)	
1	5 (3.4%)	4 (3.2%)	1 (4.2%)	
**Methodology**				**<0.001**
Hot spot	66 (44%)	62 (50%)	4 (17%)	
Sequencing	83 (56%)	63 (50%)	20 (83%)	
**Smoking Status**				**<0.001**
Never smoker	37 (25%)	22 (18%)	15 (63%)	
Smoker	112 (75%)	103 (82%)	9 (38%)	
**Smoking Status (Detailed)**				**<0.001**
Never smoker	37 (25%)	22 (18%)	15 (63%)	
Former smoker	55 (37%)	49 (39%)	6 (25%)	
Current smoker	57 (38%)	54 (43%)	3 (13%)	
**Histology Type**				0.31
ADC	123 (83%)	100 (80%)	23 (96%)	
SqCC	19 (13%)	18 (14%)	1 (4.2%)	
ASC	3 (2.0%)	3 (2.4%)	0 (0%)	
other	4 (2.7%)	4 (3.2%)	0 (0%)	
UICC Stage **				0.92
I (A–B)	82 (55%)	69 (55%)	13 (54%)	
II (A–B)	39 (26%)	32 (26%)	7 (29%)	
IIIA	28 (19%)	24 (19%)	4 (17%)	

Median +/− SD or frequency (%). Two sample *t*-test; Pearson’s Chi-squared test. * Lobectomy, segmentectomy, pneumonectomy; ** UICC, Union for International Cancer Control. ADC, adenocarcinoma; SqCC, squamous cell carcinoma; ASC, adenosquamous carcinoma. ^1^ *EGFR* wt, *EGFR* wild type; ^2^ *EGFR* mut, *EGFR* mutated. Significant *p*-values (>0.05) are highlighted in bold.

**Table 3:** Distribution of *KRAS* mutations according to patients’ features.

Feature (n%)	Overall (n = 149)	*KRAS* mut ^1^ (n = 47)	*KRAS* wt ^2^ (n = 102)	*p*-Value
**Age at Diagnosis (mm ± SD)**	67.8 ± 8.8	68.2 ± 8.8	67.1 ± 8.9	0.470
**Sex**				0.400
F	35 (23%)	9 (26%)	26 (74%)	
M	114 (77%)	38 (33%)	76 (67%)	
**Type of Surgery**				0.580
Anatomic resection *	109 (73%)	33 (30%)	76 (70%)	
Atypical resection	40 (27%)	14 (35%)	26 (65%)	
**R**				0.120
0	144 (97%)	47 (33%)	97 (67%)	
1	5 (3.4%)	0 (0%)	5 (100%)	
**Methodology**				**0.040**
Hot spot	66 (44%)	15 (23%)	51 (77%)	
Sequencing	83 (56%)	32 (39%)	51 (61%)	
**Smoking Status**				0.060
Never smoker	37 (25%)	7 (19%)	30 (81%)	
Smoker	112 (75%)	40 (36%)	72 (64%)	
**Smoking Status (Detailed)**				0.130
Never smoker	37 (25%)	7 (19%)	30 (81%)	
Former smoker	55 (37%)	18 (33%)	37 (67%)	
Current smoker	57 (38%)	22 (39%)	35 (61%)	
**Histology Type**				**0.030**
ADC	123 (83%)	44 (36%)	79 (64%)	
SqCC	19 (13%)	1 (5%)	18 (95%)	
ASC	3 (2.0%)	0 (0%)	3 (%100)	
other	4 (2.7%)	2 (50%)	2 (%50)	
UICC Stage **				0.070
I (A–B)	82 (55%)	28 (34%)	54 (66%)	
II (A–B)	39 (26%)	7 (18%)	32 (82%)	
IIIA	28 (19%)	12 (43%)	16 (57%)	

Median +/− SD or frequency (%). Two sample *t*-test; Pearson’s Chi-squared test. * Lobectomy, segmentectomy, pneumonectomy; ** UICC, Union for International Cancer Control. ADC, adenocarcinoma; SqCC, squamous cell carcinoma; ASC, adenosquamous carcinoma. ^1^ *KRAS* mut, *KRAS* mutated; *KRAS* wt, ^2^ *KRAS* wild type. Significant *p*-values (>0.05) are highlighted in bold.

## Data Availability

The raw data supporting the conclusions of this article will be made available by the authors on request.

## Supplementary Materials

The following supporting information can be downloaded at: https://www.mdpi.com/article/10.3390/cancers17050730/s1, Supplemental Table S1: Clinicopathological features of study patient cohort (n = 149); Supplemental Table S2: Subtypes of *EGFR* mutations according to patients’ features; Supplemental Table S3: Subtypes of KRAS mutations according to patients’ features.
